# Paradoxical antiproliferative effect by a murine mammary tumor-derived epithelial cell line

**DOI:** 10.1186/1471-2407-7-184

**Published:** 2007-10-01

**Authors:** Esteban N Gurzov, Sanaa M Nabha, Hamilto Yamamoto, Hong Meng, O Graciela Scharovsky, R Daniel Bonfil

**Affiliations:** 1 Department of Molecular Biology, Centro de Biología Molecular Severo Ochoa Universidad Autónoma de Madrid, Facultad de Ciencias, Madrid, Spain; 2 Departments of Urology and Pathology, Wayne State University School of Medicine and The Barbara Ann Karmanos Cancer Institute, Detroit, MI, USA; 3 Instituto de Genética Experimental, School of Medical Sciences, University of Rosario, Rosario, Argentina

## Abstract

**Background:**

Despite significant advancement in breast cancer therapy, there is a great need for a better understanding of the mechanisms involved in breast carcinogenesis and progression, as well as of the role of epigenetic contributions from stromal cells in mammary tumorigenesis. In this study, we isolated and characterized murine mammary tumor-derived epithelial and myofibroblast cell lines, and investigated the *in vitro *and *in vivo *effect of cellular soluble factors produced by the epithelial cell line on tumor cells.

**Methods:**

Morphology, immunophenotype, cytogenetics, invasiveness, and tumorigenicity of epithelial (LM-234ep) and myofibroblast (LM-234mf) cell lines isolated from two murine mammary adenocarcinomas with common ancestor were studied. The *in vitro *effects of LM-234ep conditioned medium on proliferation, cell cycle distribution, and expression of cell cycle proteins, were investigated in LM-234mf cells, mouse melanoma cells (B16-F10), and human cervical adenocarcinoma cells (HeLa). The *in vivo *anti-tumor activity of LM-234ep conditioned media was evaluated in subcutaneous tumors formed in *nude *mice by B16-F10 and HeLa cells.

**Results:**

LM-234ep cells were found to be cytokeratin positive and hipertriploid, whereas LM-234mf cells were α-smooth muscle actin positive and hypohexaploid. Chromosome aberrations were found in both cases. Only LM-234mf revealed to be invasive *in vitro *and to secrete active MMP-2, though neither of the cell types were able to produce progressing tumors. LM-234ep-derived factors were able to inhibit the *in vitro *growth of LM-234mf, B16-F10, and HeLa cells, inducing cell cycle arrest in G_0_/G_1 _phase. The administration of LM-234ep conditioned medium inhibited the growth of B16-F10 and HeLa tumors in *nude *mice.

**Conclusion:**

Our data suggest the existence of epithelial cell variants with tumor suppressive properties within mammary tumors. To our knowledge, this is the first report showing antiproliferative and antineoplastic activities induced by tumor-derived epithelial cells.

## Background

Breast cancer is the most frequent and the second deadliest neoplasm affecting females in the Western world [1]. Despite the enormous advancement in breast cancer therapy in the last years, a better understanding of the mechanisms involved in breast carcinogenesis and progression may lead to novel therapies and increased patient survival.

For decades, processes involved in malignant transformation and progression have been ascribed only to genetic alterations occurring in cells, with no influence of the surrounding microenvironment where cells were located. Because of this, most of the earlier studies were focused exclusively on cancer cells. This concept changed over the last years, as epigenetic contributions from stromal cells in close proximity to cancer cells were found to play a crucial role in the growth, angiogenesis, invasion, and metastases of most carcinomas, including those originated in breast [2-4]. In that aspect, the establishment and characterization of cell lines derived not only from neoplastic but also from stromal components of breast carcinomas are extremely important to study more accurately the biological consequences of these heterotypic cell interactions. Myofibroblasts, defined as fibroblasts with α-smooth muscle actin (SMA) expression [5], constitute the most predominant carcinoma-associated stromal cells, and have been found to be involved in the production of different proteases responsible for invasion [6-8], as well as in the stimulation of the proliferation of cancer cells [9,10].

In the present study, we succeeded at isolating epithelial (LM-234ep) and myofibroblast (LM-234mf) cell lines from two murine mammary adenocarcinomas derived from a common ancestor spontaneously arisen in BALB/c mice [11]. Despite its malignant mammary carcinoma origin, the immortalized epithelial LM-234ep cell line was not tumorigenic in either syngeneic or immunodepressed mice. Moreover, the myofibroblast LM-234mf cell line was more invasive *in vitro *than LM-234ep cell line. Paradoxically, we found that LM-234ep-derived factors were able not only to inhibit the proliferation of different cancer cells *in vitro *but also their growth *in vivo*. To our knowledge, this is the first report showing antiproliferative and antineoplastic activities induced by tumor-associated epithelial cells.

## Methods

### Establishment of LM-234 cell lines

M-234 is a mammary tumor spontaneously arisen in a BALB/c female mouse, histologically defined as a semidifferentiated carcinoma with low mitotic rate, and absence of estrogen and progesterone receptors [11]. The tumor, obtained from the Animal Care Facility of School of Medical Sciences, University of Rosario, is maintained by serial subcutaneous passages in syngeneic mice. Two tumor variants with similar characteristics obtained from M-234, namely M-234p and M-234m, were used here. Non-necrotic fragments from both tumors were used for *in vitro *culture. Briefly, palpable tumors were aseptically excised, and minced to about 1 mm^3^. The tumor pieces were disaggregated by incubation at 37°C in phosphate buffered saline (PBS) containing 0.25% bovine serum albumin (BSA), 0.25% trypsin, 0.25% collagenase type II, and 0.1% hyaluronidase (all reagents from Sigma Chemical Co., St. Louis, MO). The resulting cell suspensions were centrifuged for 10 min at 1,000 rpm, and the pellets obtained resuspended in RPMI-1640 or DMEM culture media (Sigma) with 10% fetal bovine serum (FBS, Natocor, Córdoba, Argentina). The cells derived from M-234p grew faster in RPMI-1640 than in DMEM, whereas those derived from M-234m grew only in DMEM. The cells have been maintained in culture for over 60 passages. Cell lines derived from M-234p and M-234m are referred to as LM-234ep and LM-234mf, respectively.

### Other cell lines

B16-F10, a C57BL/6 mouse melanoma cell line [12], and HeLa, a human cervix adenocarcinoma cell line [13], were both obtained from American Type Culture Collection (ATCC, Manassas, VA). They were cultured in DMEM supplemented with 10% FBS. BMA3.1A7 [14], a mouse macrophage cell line derived from bone marrow, kindly provided by Dr. Kenneth Rock (Dana-Farber Cancer Institute, Boston, MA), was cultured in RPMI 1640 with 10% FBS. The osteoblast-like cell line 7F2 [15], isolated from mouse bone marrow, was obtained from ATCC, and cultured in Alpha minimum essential medium with 2 mM L-glutamine, 1 mM sodium pyruvate without ribonucleosides and deoxyribonucleosides, supplemented with 10% FBS.

### Immunocytochemistry and immunofluorescence analyses

LM-234ep and mf cells were grown overnight on sterile 13-mm Thermanox™ coverslips or Lab Tek™. 4-chamber glass slides, both from Nunc (Naperville, IL). Cells were rinsed in PBS, and then fixed in ice-cold methanol (-20°C) for 30 min. After rinsing in PBS, immunoreactive proteins were detected by one of three methods: 1) for detection of SMA, slides were incubated with a fluorescein-conjugated monoclonal antibody that cross reacts with human and mouse αSMA (20 μg/ml; Biomeda, Foster City, CA) for 1 h at room temperature, and then counterstained with DAPI (Sigma, 5 μg/ml Tris buffer) for 20 sec, and mounted with Vectashield (Vector Laboratories, Burlingame, USA); 2) for protein detection, slides were incubated for 1 h at 37°C with a 1:200 antibody dilution of mouse anti-c-Jun (Transduction Laboratories, San Diego, CA), rabbit anti-cyclin A or rabbit anti-cyclin D (Santa Cruz Biotechnology, Inc., Santa Cruz, CA), and then incubated for another hour with an anti-mouse or rabbit FITC-antibody; 3) for detection of cytokeratin, endogenous peroxidase activity was quenched with 3% hydrogen peroxide in methanol, and non-specific immunobinding blocked with Superblock (Scytek Laboratories, Logan, UT) for 10 min at room temperature. Slides were then incubated with an anti-Pan cytokeratin antibody (1:50; Sigma) for 2 h at room temperature. After rinsing with PBS, Vectastain Elite ABC kit for mouse IgG (Vector Laboratories) was used. Immunoreactive cytokeratins were recognized as a brown color after incubation with diaminobenzidine (DAB; Sigma)/H_2_O_2 _solution. Slides were lightly counterstained with Mayer hematoxylin before mounting. For each epitope analyzed, species/isotype-matched pre-immune IgGs served as negative primary antibody controls.

### Karyotyping

Exponentially growing LM-234ep and LM-234mf cell cultures were exposed to 0.2 μg/ml colcemid (Sigma) for one hour at 37°C. Metaphase spreads were prepared and stained by conventional methods as described previously [16]. A minimum of fifty metaphases for each cell line was selected and photographed to determine chromosome frequency distribution and morphology.

### Zymography

Confluent monolayers of LM-234mf and LM-234ep cells were incubated for 48 h in FBS-free culture media containing 0.1% BSA, to obtain conditioned media (CM). All CM were cell number-normalized. The gelatinase activity was detected by zymography in 10% sodium dodecyl sulfate-polyacrylamide gels containing 1 mg/ml gelatin (Sigma), as described previously [17]. Gelatinolytic bands were visualized as transparent areas against the dark-blue background. As positive controls for murine MMP-2 and MMP-9, CM derived from 7F2 and BMA3.1A7 cell lines were used, respectively.

### Chemoinvasion assay

Cell invasion assay was carried out in Transwell^® ^cell culture chambers (Corning Costar, Cambridge, Massachusetts) essentially as previously described [18]. Briefly, the upper surface of the insert membrane (8-μm pore size) was coated with 37.5 μg/100 μl of cold reconstituted basement membrane Matrigel™, and dried overnight at room temperature in a tissue culture hood. The lower compartment of the Transwell^® ^contained 5% FBS, which was used as chemoattractant. LM-234ep or LM-234mf cells were seeded at a density of 1 × 10^5^per Transwell^® ^culture insert in 100 μl of DMEM containing 0.1% BSA. The chambers were incubated at 37°C for 18 h. Non-migrating cells on the upper surface of the filters were removed with a cotton swab, whereas the cells that traversed the filters were stained with Diff-Quick (Dade-Behring, Newark, DE), and counted in 10 random 20× fields using a light microscope. All the assays were carried out in triplicate.

### Tumorigenic potential

Subconfluent LM-234ep and LM-234mf cell cultures were trypsinized and resuspended in their respective FBS-free culture medium. The viability of both cell types was above 90%, as determined by trypan blue exclusion. To test the tumorigenicity of the cell lines, 1 × 10^6^-1 × 10^7 ^cells were subcutaneously or intramammary fat pad inoculated into 10–12 week-old female BALB/c (from the Rodent Facility of the University of Rosario School of Medical Sciences) or 8–12 week-old congenitally athymic female Swiss *nude *mice (from the Mice Facility of Centro de Biología Molecular Severo Ochoa, Universidad Autónoma de Madrid). Co-inoculation of cells and Matrigel™ (BD Biosciences, Rockville, MD), a basement membrane preparation extracted from EHS mouse sarcoma, was performed in some cases in an attempt to enhance tumorigenicity of the cells [19]. To study the effect of heterotypic interaction on tumor growth, experiments involving co-inoculation of LM-234mf and LM-234ep cells (1.5 × 10^6 ^cells each) in *nude *mice were performed. Tumors volumes were calculated as: *π/6 *× *Length *× *Width *× *Height*, based on periodic measurement on calipers. *Nude *mice were maintained under aseptic conditions.

### Co-culture studies

LM-234ep and LM-234mf cells were seeded at 1:1 ratio (10^4 ^cells for each cell line) onto Thermanox™ coverslips that were placed inside 24-well tissue culture plates. Cells were co-cultured in DMEM supplemented with 10% FBS, and coverslips were harvested 4 and 8 days after cell seeding for cytokeratin immunocytochemistry, performed as explained above. Digital photomicrographs were captured using a Zeiss Axioplan 2 microscope (Zeiss, Göttingen, Germany) equipped with a software-controlled (Axiovision, Zeiss) digital camera.

### Effect of conditioned media on cell proliferation

LM-234mf, B16-F10, and HeLa cells were seeded into 6-well plates at a density of 1 × 10^4 ^cells/well on day 0, and allowed to attach overnight. Thereafter, cells were incubated in the presence of 1 ml of either LM-234ep or LM-234mf CM, obtained after culturing confluent monolayers for 48 h in their culture media containing 10% FBS, or their respective complete culture medium (control). To avoid non specific effect of the LM-234ep or LM-234mf CM, the pH was controlled in all assays, the glucose, lactate and glutamine were measured using a Biochemistry analyzer YSI 2700 Select (YSI Inc., Yellow Springs, OH) and we have used the same cell numbers of LM-234ep in all the experiments to obtain the CM. Cell numbers were counted every 2 days up to day 7 with a Neubauer chamber.

### Fluorescence-activated cell sorting (FACS) analysis

After being exposed to LM-234ep CM or culture medium, both supplemented with 10% FBS, LM-234mf, B16-F10, and HeLa cells were harvested by trypsin digestion, resuspended in cold PBS, and fixed in 75% ethanol at 4°C for at least 8 h. After a brief centrifugation, fixed cells were resuspended in PBS containing propidium iodide (5 μg/ml; Sigma)/RNase A (30 μg/ml, Sigma). After incubation for 30 min at room temperature, DNA staining was evaluated by fluorescence 2 intensity in the linear scale using FACScan.

### Immunoblotting

Cells were lysed on ice in lysis buffer (50 mM Tris-HCl (pH 7.5), 300 mM NaCl, 0.5% Triton ×-100, 1 mM EDTA and protease inhibitor). Cellular debris was removed by centrifugation of lysates for 10 min at 14,000 g. The supernatant was collected, and equal amounts of proteins, as determined by the BCA protein micro assay (Bio-Rad Laboratories, Hercules, CA), were resolved by sodium dodecyl sulfate-polyacrylamide gel electrophoresis and electroblotted onto nitrocellulose membranes. After the transfer, membranes were blocked with a 10% (wt/vol) solution of skim milk powder in PBS, and then probed with one of the following primary antibodies: rabbit anti-p-ERK (1:200; Santa Cruz Biotechnology), mouse anti-c-Jun (1:1,000; Transduction Laboratories), rabbit anti-JunB (1:500; Santa Cruz Biotechnology), rabbit anti-cyclin E (1:200; Santa Cruz Biotechnology), rabbit anti-cyclin A (1:200; Santa Cruz Biotechnology), rabbit anti-cyclin D (1:200; Santa Cruz Biotechnology), mouse anti-Cdk2 (1:1,000; BD Transduction Laboratories), or rabbit anti-β-actin (1:200, Sigma). The different antigens were detected by chemiluminescence, using horseradish peroxidase-conjugated antibody (1:500; Dako, Carpinteria, CA).

For immunodetection of cytokeratins, membranes were incubated with a 1:500 dilution of monoclonal anti-pan cytokeratin (Sigma), which recognizes cytokeratins 1, 4, 5, 6, 8, 10, 13, 18 and 19. The cytokeratins detected by these antibodies range in molecular weight between 40 kDa and 68 kDa. For immunodetection of αSMA a monoclonal antibody (clone 1A4, Sigma) was used at 1:200.

### Measurement of AP-1 activity

Cells were transfected with the AP-1-luciferase reporter gene plasmid carrying the collagenase promoter (p-73col-luc) [20] or with the control plasmid without the AP-1 site and the pRL-tk-luc plasmid (Promega, Madison, WI) to normalize for transfection efficiency. After 24 h, the activity was determined using a Promega luciferase assay kit following the manufacturer's protocol. Data were normalized to *Renilla *luciferase activity and are presented in relative luciferase units. Transfections were always run in triplicate and data presented as mean ± SD.

### Treatment of tumor-bearing mice with LM-234ep conditioned medium

*Nude *mice were subcutaneously injected with 5 × 10^5 ^B16-F10 or 1 × 10^6 ^HeLa cells in both flanks. Tumors were allowed to grow until they became palpable, and then locally injected with 200 μl of FBS-free LM-234ep CM in the left flank, and with an equal volume of FBS-free culture medium contralaterally. Treatments were performed once every 2 days in mice injected with B16-F10 cells, and once a week in mice injected with HeLa cells. Treatments were continued until tumors reached a median volume of 500 mm^3^. Five mice per group were used.

### Histological analysis and immunohistochemistry

Tumors were fixed in 10% buffered formaldehyde, paraffin-embedded, and processed by routine methods to get 5-μm consecutive histological sections that were used for H&E staining and immunohistochemical studies. To determine the proliferative activity within the tumors, sections were pretreated in Ag Citrus Plus Retrieval Solution (BioGenex, San Ramon, CA) in a microwave, and then incubated with anti-Ki67 monoclonal antibody (1:40; BioGenex, San Ramon, CA). Intratumoral microvascularity was assessed using a rat anti-mouse CD34 antibody (1:25; Cell Sciences, Canton, MA). Mouse on Mouse (MOM™) immunodetection peroxidase kit (Vector, Burlingame, CA) and Vectastain Elite ABC kit for rat IgG (Vector Laboratories) were used to visualize Ki-67 and CD34 immunoreactive sites, respectively. Slides were lightly counterstained with Mayer hematoxylin before mounting. In each case, species/isotype-matched pre-immune IgGs served as negative primary antibody controls.

### Statistical analysis

Data were statistically analyzed using Student's *t *test using GraphPad InStat^® ^version 3.0 (GraphPad Software, San Diego, CA). Differences were considered to be statistically significant at *P *< 0.05.

## Results

### Morphology and immunophenotypic analysis

**Figure 1:**
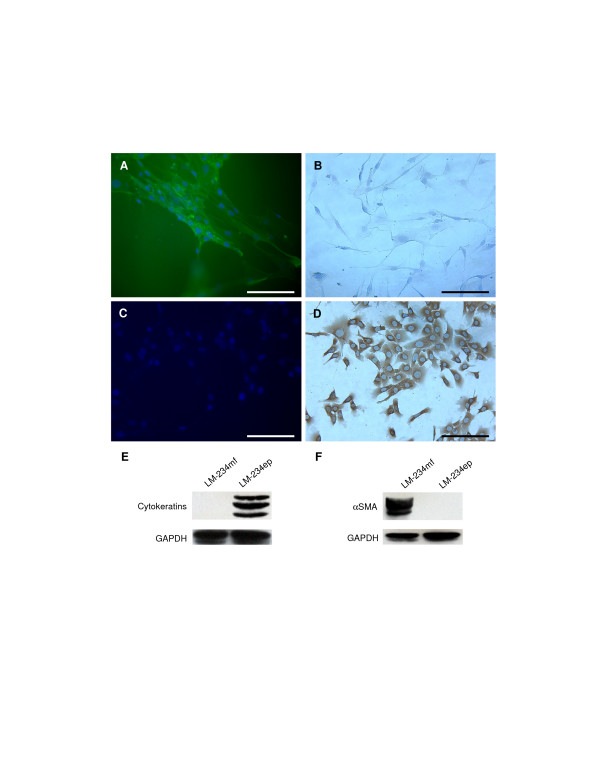
**Alpha smooth muscle actin (SMA) and cytokeratin expression**. SMA (green staining) was detected in LM-234mf (A) but not in LM-234ep (C) cells, using a FITC-conjugated primary antibody. Nuclei (blue staining) were identified using DAPI. Cytokeratin was undetectable in LM-234mf (B), whereas strongly expressed in LM-234ep (D), as revealed by immunohistochemistry using an anti-Pan cytokeratin antibody. Magnification bar, 100 μm. Cell lysates (25 μg/lane) were analyzed using Western blot for cytokeratins (E) and αSMA (F). The cytokeratins detected with the anti-pan antibody used range between 40 and 68 kDa. The band immunodetected for αSMA has an approximate molecular weight of 42 kDa. Glyceraldehyde 3-Phosphate Dehydrogenase (GAPDH) was used as a loading control.

LM-234ep and LM-234mf cells grew in an adherent fashion and have been maintained in culture for more than 60 passages. Their *in vitro *doubling time, calculated from the logarithmic phase (days 1 to 5) of growth, was 25 h for LM-234ep and 20 h for LM-234mf cells. Immunocytochemical and immunofluorescence analysis for SMA and cytokeratin showed that LM-234mf cells expressed SMA but not cytokeratin (Figures 1A and 1B, respectively), whereas LM-234ep cells expressed cytokeratin only (Figures 1C and 1D). These results, confirmed by immunoblotting (Figures 1E and 1F), indicate that LM-234mf cells exhibit characteristics of myofibroblasts, while LM-234ep cells have an epithelial origin. From a morphological point of view, LM-234mf cells were spindle-shaped, revealing an intermediate phenotype between fibroblasts and smooth muscle cells. Conversely, LM-234ep cells were epitheloid.

### Chromosome analysis

**Figure 2:**
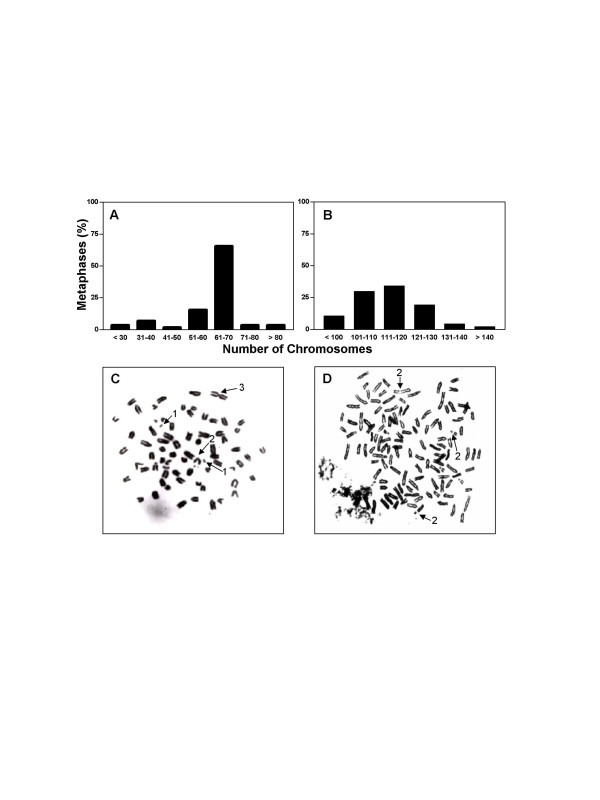
**Chromosome analysis of LM-234 cell lines**. Histograms showing the chromosome number distribution in LM-234ep (A) and LM-234mf (B) cells. A minimum of 50 metaphase spreads was analyzed in each case. Representative metaphases of LM-234ep (C) and LM-234mf (D) cells. Arrows point different chromosomal aberrations including: acentric fragments (1), chromosome break (2), and centromeric fusion (3). Magnification, × 1000.

The cytogenetic analysis revealed aneuploidy for both cell lines (Figures 2A and 2B). While the normal mouse karyotype consists of 40 acrocentric chromosomes, more than 60% of the LM-234ep cells showed between 61 and 70 chromosomes, with a modal number of 63 (Figure 2A). As for LM-234mf cells, more than 70% of the cells had between 101 and 130 chromosomes, with a modal number of 111 (Figure 2B). The near-triploid (LM-234ep) and near-hexaploid (LM-234mf) patterns shown might result from endoreduplication/s occurred after some chromosomal rearrangements. Chromosome aberrations found in both cases include acentric fragments, chromosome breaks, and centromeric fusions (Figures 2C and 2D).

### Differential in vitro gelatinase production and invasive capacities

**Figure 3:**
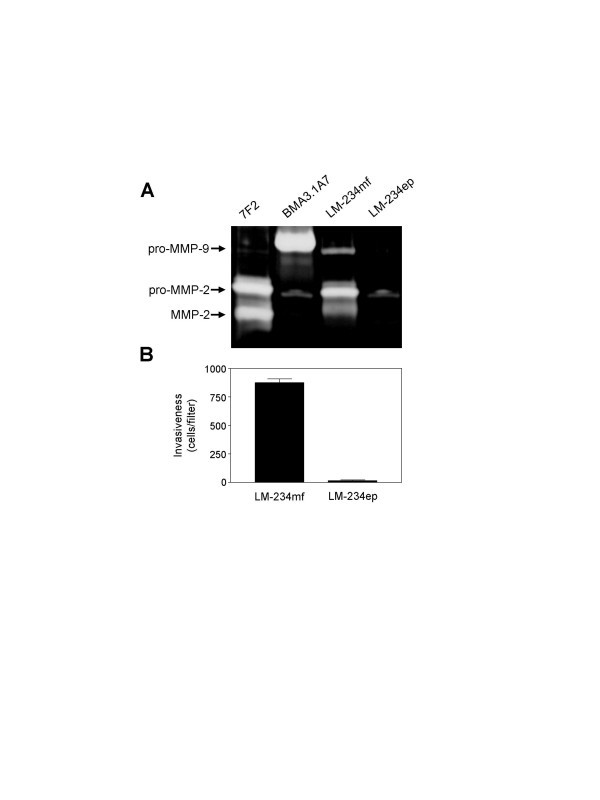
**Gelatinase expression/activity and invasive abilities of LM-234 cell lines**. Conditioned media derived from LM-234mf and LM-234ep cells were analyzed by gelatin zymography, using 7F2 and BMA3.1A7 cell lines as positive controls for mouse MMP-2 and MMP-9, respectively (A). LM-234mf and ep cells were compared for their ability to invade through Matrigel-coated Transwell 8 μm-pore filters (B). The number of cells traversing the membrane was quantified and expressed as mean ± SE (n = 3).

FBS-free CM obtained from both cell types were analyzed using gelatin zymography to determine whether MMP-2 and MMP-9 were produced and secreted as zymogens and/or active species. As can be seen in Figure 3A, LM-234mf cells secreted MMP-9 in its latent form, as well as MMP-2 as zymogen and an intermediate active species with higher molecular weight than the fully active MMP-2. LM-234ep cells only secreted pro-MMP-2 in relatively less amount than LM-234mf cells (Figure 3A). We also used an *in vitro *reconstituted basement membrane invasion assay to determine the invasive capacity of LM-234ep and LM-234mf cells. We found that LM-234mf cells were highly invasive, as opposed to the tumor-derived epithelial LM-234ep cell line (Figure 3B). These results show a clear association between expression of active MMP-2 and invasiveness in these cell lines.

### Tumorigenicity

The tumorigenic capacity of both LM-234 cell lines was tested between passages 15 to 20 and between passages 45 to 50. Subcutaneous and intramammary fat pad inoculations of 10^6^, 5 × 10^6^, and 10^7^cells were performed in syngeneic BALB/c mice. LM-234ep cells were capable to generate palpable growths that peaked by day 4 after inoculation of 10^7 ^cells, although their average median volume was only 5 mm^3 ^and they regressed completely by day 8. On the contrary, LM-234mf cells never gave rise to clinically detectable tumors for more than 3 months of observation. The same results were obtained with both cell lines when subcutaneously or intramammary fat pad injected into *nude *mice, or co-inoculated with Matrigel™, independently of the passage number (data not shown). The effect of co-inoculation of LM-234mf and LM-234ep cells on tumor growth was also measured. Four days after inoculation, when tumescence peaked, volumes were larger in mice co-injected with LM-234mf and LM-234ep (33.6 ± 3.9 mm^3^) than in mice injected with LM-234ep cells alone (4.9 ± 1.9 mm^3^). However, all the growths regressed by day 12. Mice injected with LM-234mf cells alone did not develop any growths.

### Paracrine effects in crossover experiments

**Figure 4:**
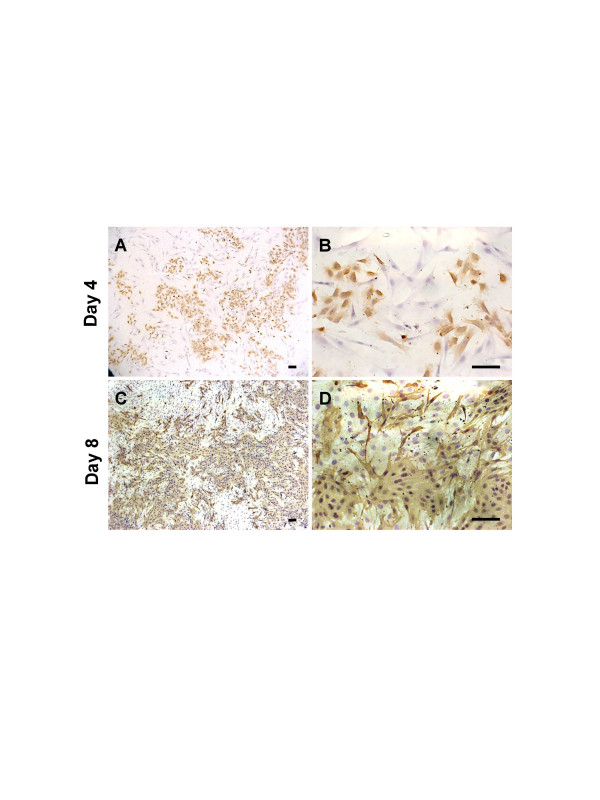
**LM-234ep cells overgrow LM-234mf cells**. Cells were co-cultured at a 1:1 ratio and immunostained for cytokeratin on days 4 (A and B) and 8 (C and D) to distinguish LM-234ep (cytokeratin positive) from LM-234mf cells. Note the predominance of LM-234ep over LM-234mf cells on day 8. Magnification bar, 100 μm.

To study the effect of *in vitro *cell-cell interaction, LM-234ep and LM-234mf cells were co-cultured at 1:1 ratio in DMEM supplemented with 10% FBS. Four and eight days after seeding, cells were immunostained for cytokeratin to distinguish LM-234ep from LM-234mf cells. Despite longer doubling time of LM-234ep cells, slower growth of these cells when cultured in DMEM, and a similar distribution of both cell types on day 4 (Figures 4A and 4B), LM-234ep cells finally outgrew LM-234mf cells by day 8 (Figures 4C and 4D).

**Figure 5:**
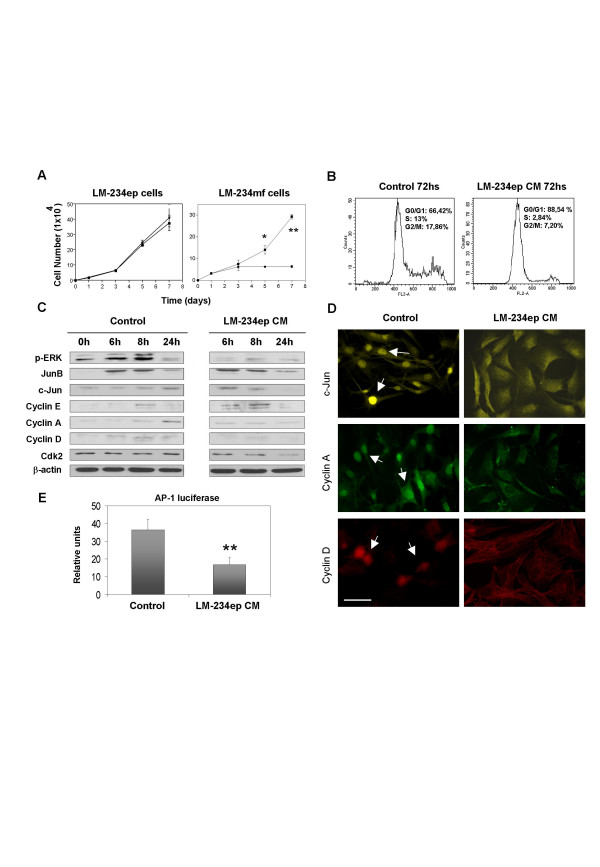
**Inhibitory effect of LM-234ep conditioned medium on the proliferation of LM-234mf cells**. LM-234mf and LM-234ep cells were seeded into 6-well plates at a density of 1 × 10^4 ^cells/well and allowed to attach overnight. Thereafter, cells were incubated in the presence of LM-234ep CM (black circles), LM-234mf CM (black triangles), or culture medium (black squares) supplemented with 10% FBS. Cell numbers were counted at different time points, and expressed as mean ± SE. * *P *= 0.05, ** *P *= 0.0018 (Student's t Test). Assays were performed in triplicate, (A). LM-234mf cells were incubated with culture medium + 10% FBS (Control) or LM-234ep CM for 72 h. Cells were processed for DNA content, and cell cycle progression was analyzed by flow cytometry (B). LM-234mf cells were treated with culture medium (Control) or LM-234ep CM for the times indicated (top). Cells were collected, lysed, and analyzed by immunoblotting using antibodies specific for p-ERK, c-Jun, JunB, cyclins E, A, and D, and Cdk2. β-actin was used as a loading control (C). Immunofluorescence for c-Jun, Cyclin A and Cyclin D in LM-234mf cells. The nuclear localization of the proteins is shown in the control cells (white arrows). Magnification bar, 40 μm (D). AP-1 luciferase activity in LM-234mf cells co-transfected with AP-1-Luc and pRL-tk-luc and treated with LM-234ep CM or culture medium (Control). ** *P *= 0.005 (Student's t Test). The results shown are the mean ± S.D. of three experiments (E).

Crossover experiments, in which FBS-containing CM of each of the cell lines was tested on the growth of the other cell line, were also performed. Surprisingly, CM from LM-234ep cells caused a significant inhibition of LM-234mf cell growth, as opposed to LM-234mf CM, which did not alter the growth curve of LM-234ep over a period of 7 days (Figure 5A). FACS analysis of the cell cycle in LM-234mf cells treated with LM-234ep CM, showed a significant increase in the G_0_/G_1 _population, with a concomitant decrease in the S phase and G_2_/M populations in all cases, as compared with cells only treated with culture medium (Figure 5B). Importantly, no apoptotic cells were found in the subG_0 _phase of the cell cycle, which indicates that LM-234ep CM induced cell growth arrest but not cell death. These data support the results obtained in the co-culture studies where cell-cell contact exists, and suggest an inhibitory effect of LM-234ep on LM-234mf.

The ERK/activating protein 1 (AP-1) pathway is implicated in fundamental cellular processes including differentiation, cell proliferation and oncogenic transformation [21,22]. Since CM from LM-234ep cells caused inhibition of LM-234mf cell growth, we analyzed the changes of p-ERK and JunB/c-Jun proteins after the addition of the LM-234ep CM in G_0_-synchronized and serum-stimulated LM-234mf cells using Western blot. As shown in Figure 5C, the levels of p-ERK and c-Jun were decreased in the cells treated with LM-234ep CM, while JunB expression remained unchanged. Immunofluorescence analyses confirmed the c-Jun inactivation (Figure 5D). We next analyzed AP-1 activity in LM-234mf cells upon LM-234ep CM treatment using a reporter assay. Reduced levels of p-ERK/c-Jun led to a significant decrease in AP-1 activity, as measured using a collagenase promoter reporter construct (Figure 5E). Previous data demonstrated that AP-1 inactivation induced down-regulation of cell cycle molecules in breast cancer cells [23]. As compared to control cells exposed to culture medium, LM-234mf cells incubated with LM-234ep CM resulted in an evident densitometric decrease in the expression of cyclin A, D, and Cdk2, whereas the expression of cyclin E did not change (Figures 5C). Reduction in Cyclin A and D expression was confirmed in the cells treated with LM-234ep CM by immunofluorescence (Figures 5D).

### Antiproliferative effect of LM-234ep conditioned media on tumor cell lines

**Figure 6:**
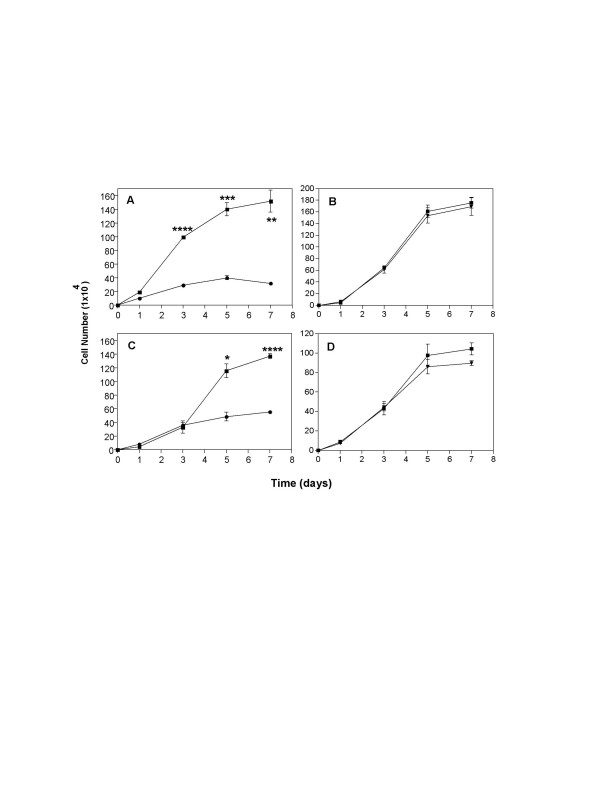
**LM-234ep conditioned medium inhibits cell proliferation in cancer cells**. B16-F10 (A and B), or HeLa (C and D) cells were seeded into 6-well plates at a density of 1 × 10^4 ^cells/well and allowed to attach overnight. Cells were incubated with LM-234ep CM (black circles), LM-234mf CM (black triangles), or culture medium (black squares) containing 10% FBS (Control), and counted at different time points. Data are shown as mean ± SE. * *P *= 0.05, ** *P *= 0.0018, *** *P *= 0.0006, **** *P *< 0.0001 (Student's t Test). Assays were performed in triplicate.

**Figure 7:**
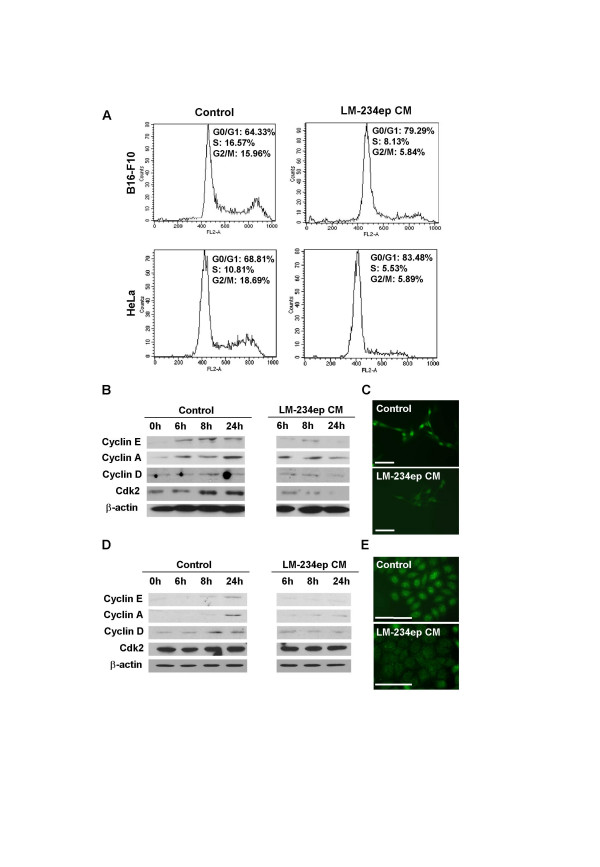
**Effects of LM-234ep conditioned medium on cell cycle and its regulatory proteins**. B16-F10 and HeLa cells were treated with culture medium (Control) or LM-234ep CM for 48 h. Cell cycle progression was analyzed by flow cytometry (A). After the treatment for the indicated times (top) with LM-234ep CM or culture medium (Control), B16-F10 (B) and HeLa (D) cells were collected, lysed, and analyzed by immunoblotting using antibodies specific for cyclins E, A, and D, and Cdk2. β-actin was used as a loading control. Immunofluorescence for Cyclin A1 in B16-F10 (C), and HeLa (E) cells after 24 h incubation with culture medium or LM-234ep CM. Magnification bar, 40 μm.

The effect of LM-234ep CM was also tested on the *in vitro *proliferation of the mouse melanoma-derived B16-F10 cell line and the human cervix adenocarcinoma-derived HeLa cell line. Cells were allowed to attach overnight, and then incubated with FBS-containing CM from LM-234ep and LM-234mf cells for 7 days. As can be seen in Figures 6A and 6C, a potent inhibition on the proliferation of both cancer cell types was obtained after treatment with LM-234ep CM despite the presence of growth factors in FBS. Conversely, no significant inhibition on the proliferation of either of the tumor cell lines tested was observed when LM-234mf CM was employed (Figures 6B and 6D). The analysis of DNA content indicates that the proliferation arrest is also induced in the G_0_/G_1 _phase of the cell cycle in these cells (Figure 7A). Reduction in the expression of cyclin A, D, E, and Cdk2 was observed in B16-F10 cells exposed to LM-234ep CM (Figure 7B), while a down-regulated expression in cyclin A, D and E, and no change in Cdk2, were detected in HeLa cells (Figures 7D). Immunofluorescence analysis indicated a negative nuclear localization of cyclin A in B16-F10 and HeLa cells after LM-234ep CM treatment (Figure 7C and 7E). We also tested the relevance of the estrogen receptor (ER) in the cell arrest induced by the LM-234ep CM using ER+ (MCF-7) or ER- (MDA-31) breast cancer cell lines. We found a significant inhibition of the proliferation in both cell types by LM-234ep CM but not by LM-234mf CM, suggesting that the inhibitory effect shown by LM-234ep CM is ER-independent (data not shown).

### Inhibition of in vivo tumor growth by LM-234ep conditioned medium

**Figure 8:**
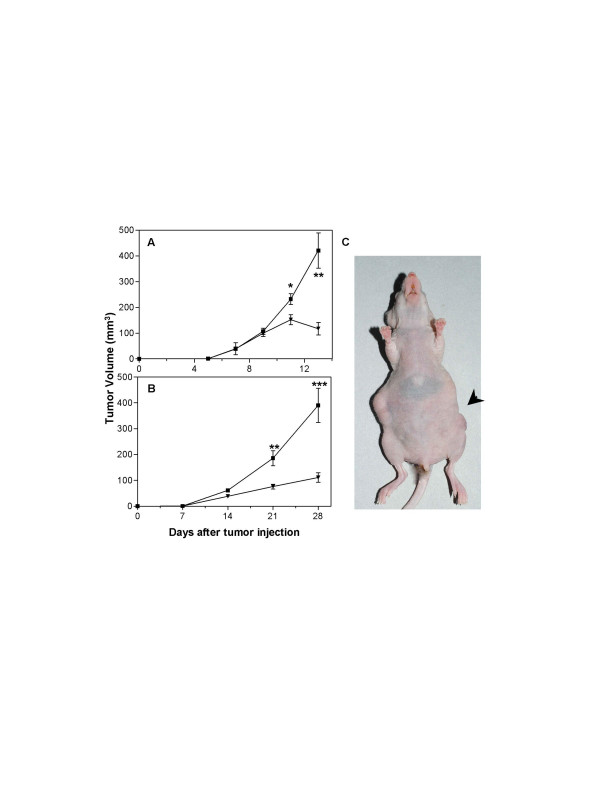
**Inhibition of *in vivo *tumor growth by LM-234ep conditioned medium**. B16-F10 (A) and HeLa (B) tumors growing in the right flank were injected with serum-free culture medium (black squares), whereas tumors in the left flank were injected with LM-234ep CM (black triangles). Tumors were measured and volumes were calculated as mean ± SE. * *P *= 0.018, ** *P *= 0.007, *** *P *= 0.004 (Student's t Test). Representative picture of a mouse showing inhibition of HeLa tumor growth by LM-234ep CM (arrow) as compared to culture medium treatment (contralateral flank), 4 weeks after tumor inoculation (C).

Finally, we evaluated the effect of FBS-free conditioned medium derived from LM-234ep cells on the *in vivo *growth of B16-F10 or HeLa subcutaneous tumors in *nude *mice. When tumors became palpable, local treatments with FBS-free CM derived from LM-234ep cells (left flank) and culture medium (right flank) were initiated. As soon as 6 and 7 days after treatment began, a significant inhibition of growth was evident in B16-F10 (Figure 8A) and HeLa (Figures 8B and 8C) tumors treated with LM-234ep CM, as compared to contralateral tumors injected with culture medium.

Tumor angiogenesis was evaluated on paraffin-embedded sections using CD34 immunostaining. Microvessel densities per 20× field in hot spots were similar in HeLa tumors treated with LM-234ep CM and regular culture medium (28.8 ± 3.3 and 28.2 ± 4.2 CD34+ structures, respectively). However, immunohistochemical staining of the tissue sections by Ki-67 revealed a significant lower proliferation index in LM-234ep CM-treated than in culture medium-treated tumors (361.5 ± 28.0 and 474.3 ± 27.0 Ki67+ cells/mm^2^, respectively, *P *< 0.05). Similar results were found in B16-F10 tumors growing in *nude *mice following the same treatment. These results suggest that the antineoplastic effect of LM-234ep CM is due to a direct antiproliferative effect on the tumor cells rather than to an inhibition of their angiogenesis.

## Discussion

For many decades, the neoplastic phenotype has been considered to be driven only by genetic alterations occurring in cells. However, many studies performed in the last years have demonstrated that genetic changes in individual cells may not be sufficient, and that the cellular microenvironment has a powerful influence in determining the final fate of cells in the process of transformation [24-26]. The oncogenic potential of cells can be modulated by adjacent cells in opposite directions, sometimes contributing to tumor formation [4,27] and others impeding it [28,29]. Moreover, stromal cells present in the tumor microenvironment have a profound effect on tumor growth, invasion and metastatic progression [30]. This is also true for breast cancer, where most of the studies have focused on the luminal epithelial cells found in the ducts and alveoli, despite the presence of stromal cells including fibroblasts, myofibroblasts, leukocytes, and myoepithelial cells [4,27]. Myofibroblasts, for instance, which are usually absent in normal tissue derived from breast, have been associated with tumor progression [26,31].

To attain a better understanding of the complex heterotypic interactions that occur in breast cancer, suitable model systems should involve cells representing both epithelial and stromal cells ideally derived from mammary tumors. Here, we isolated and established two cell lines from mammary carcinomas derived from a common tumor spontaneously arisen in a BALB/c female mouse [11]. On the basis of their immunophenotypical features, the cytokeratin positive and SMA negative LM-234ep cell line was considered of epithelial origin, whereas LM-234mf, which is positive for SMA but lacks cytokeratin expression, was categorized as myofibroblast. Both cell lines have been growing in culture for over 60 passages, with abnormalities in the number and structure of their chromosomes. In the near-triploid LM-234ep cells, double-minutes are found, suggesting genomic amplification. As for the near-hexaploid LM-234mf cells, mostly chromosomes breaks are observed. Despite these features characteristic of cancerous cells, both cell lines did not generate progressing tumors mice when different numbers of cells were injected subcutaneously (with or without Matrigel) or orthotopically in the mammary fat pad of either syngeneic mice or *nude *mice. Due to their epithelial phenotype, their immortalized nature, and the fact that they were derived from a mammary tumor, LM-234ep cells appear to derive from carcinoma cells, though they only revealed an incipient growth *in vivo *that did not persist. Although some ER positive breast cancer cells do not form tumors unless the hosts are treated with β-estradiol, that would not be the case for LM-234ep cells that are ER negative (data not shown). As for LM-234mf myofibroblast cells, it is not clear why they escaped from senescence and revealed anomalous chromosome patterns usually present in cancerous cells, but did not induce tumor formation. These results agree with those obtained in an immortalized myofibroblast cell line obtained from a human liver angiosarcoma, which also shows severe chromosomal alterations and inability to induce tumors [32]. Particularly in breast cancer, where epithelial-mesenchymal transition occurs in approximately 18% of tumors [33], a transdifferentiation of epithelial tumor cells into myofibroblasts that lose their malignant phenotype has been reported [34]. We hypothesize that this could have been the case for LM-234mf myofibroblasts, which are not tumorigenic but show aneuploidy, characteristic of cancer cells. In fact, myofibroblasts are considered a desmoplastic reaction that may facilitate mammary tumor growth and invasion [34]. Myofibroblasts, for example, can invade certain tumors preceding endothelial invasion necessary for angiogenesis [35,36]. Here, we show that LM-234mf cells are highly invasive *in vitro *and secrete MMP-2 in an active form, whereas epithelial LM-234ep cells are almost non invasive and only secrete MMP-2 as a zymogen.

It is now widely accepted that a concerted action of epithelial and stromal cells is necessary to support tumor growth and progression. Having two different cell types derived from murine mammary tumors of common origin, we aimed at elucidating the impact of their heterotypic interaction on tumor growth. As we mentioned above, only LM-234ep cells induced a small and brief tumescence *in vivo *that then regressed. Despite an enhanced growth obtained when LM-234mf and LM-234ep cells were co-inoculated in *nude *mice, this ended up in a complete regression. When both cell types were co-cultured, a predominance of LM-234ep over LM-234mf was evident. This cannot be explained in terms of different growth rates, as LM-234ep cells have a longer doubling time than LM-234mf cells. Taken together, our results suggests an inhibitory effect of LM-234ep on LM-234mf cells, and could explain why the initial enhancing effect revealed by LM-234mf on LM-234ep cell growth *in vivo *was finally hampered by the epithelial cell type. This inhibitory effect displayed by LM-234ep cells would be accomplished by diffusible factors, since LM-234ep CM revealed strong inhibitory activity *in vitro *on LM-234mf cell proliferation, which would be mediated by down-regulation of the ERK/AP-1 pathway. The antiproliferative effect was also observed on a mouse melanoma cell line (B16-F10) and on a human cervix adenocarcinoma (HeLa) and, in both cases, is the result of an arrest of the cells at the G_0_/G_1 _phase of the cell cycle. The reduced levels of cyclins E, A, and D, and of Cdk2 would account for the observed decrease in the percentage of cells in the S-G_2_/M phase. These results suggest that the inhibitory factor(s) derived from LM-234ep cells can directly or indirectly down-regulate the expression of those cell cycle genes. Moreover, the growth of tumors formed in *nude *mice by injection of HeLa and B16-F10 cells was inhibited by local treatment with LM-234ep CM. The immunohistochemical analysis of the nuclear Ki67 proliferation marker suggests an antiproliferative direct effect on the tumor cells.

Different studies have demonstrated that the normal myoepithelium can function as an inhibitor of growth, angiogenesis, invasion, and metastasis of breast cancer cells [37-40]. However, to the best of our knowledge, there are no reports on growth inhibitory activity of soluble factors derived from tumor-associated epithelial cells. Our results suggest that within the highly heterogeneous mammary tumor cell populations there are epithelial cells acting as guardians that may counterbalance malignant progression through antiproliferative effects. Further studies are currently in progress to enlighten the nature of the factor(s) produced by tumor-derived LM-234ep that are responsible for the inhibitory effect demonstrated on tumors.

## Conclusion

The results herein obtained indicate that LM-234ep and LM-234mf cell lines are suitable models to examine several aspects of tumor biology, in particular those related to the different pathways involved in the genetic events leading to tumor generation. We consider both cell lines as transformed but non tumorigenic. Also, as these cell lines are neither normal nor malignant, and can be situated somewhere in between those two stages, they could be used as a model to study different therapeutic strategies aiming to revert the transformed immortal cell type to a normal senescent one.

More interestingly, our data suggest the existence of epithelial cells with tumor-inhibitory activity within mammary tumors. The LM-234ep cell line characterized here would exemplify such cells that are transformed, as they displayed several chromosomal anomalies and did not senesce when cultured *in vitro*, and could represent a protective mechanism against tumor progression. The broad inhibitory spectrum activity demonstrated by the factor(s) secreted by LM-234ep cells on the *in vitro *proliferation and the *in vivo *growth of two non-related tumors justifies further studies that could derive in a novel therapeutic option for cancer.

## Abbreviations

BSA, bovine serum albumin; Cdk2, cyclin-dependent kinase 2; CM, conditioned medium; DAB, diaminobenzidine; EMT, epithelial-mesenchymal transition; ER, estrogen receptor; FACS, fluorescence activated cell sorting; FBS, fetal bovine serum; GAPDH, Glyceraldehyde 3-Phosphate Dehydrogenase; PBS, phosphate buffered saline; SMA, α-smooth muscle actin; TUNEL, deoxynucleotidyltransferase-mediated dUTP-biotin nick end labeling

## Competing interests

The author(s) declare that they have no competing interests.

## Authors' contributions

ENG carried out most of the studies, drafted the manuscript, and participated in the analysis and interpretation of data. SN optimized the gelatin zymographic studies and proliferation assays, and participated in the analysis and interpretation of data. HY and HM performed the immunohistochemical and proliferation assays. GS and DB conceived and coordinated the study, participated in the analysis and interpretation of data, and helped to draft the manuscript. All authors read and approved the final manuscript.

## Pre-publication history

The pre-publication history for this paper can be accessed here:



## References

[B1] Jemal A, Siegel R, Ward E, Murray T, Xu J, Smigal C, Thun MJ (2006). Cancer statistics, 2006. CA Cancer J Clin.

[B2] Ronnov-Jessen L, Petersen OW, Bissell MJ (1996). Cellular changes involved in conversion of normal to malignant breast: importance of the stromal reaction. Physiol Rev.

[B3] Skobe M, Fusenig NE (1998). Tumorigenic conversion of immortal human keratinocytes through stromal cell activation. Proc Natl Acad Sci U S A.

[B4] Bissell MJ, Radisky D (2001). Putting tumours in context. Nat Rev Cancer.

[B5] Ronnov-Jessen L, Petersen OW, Koteliansky VE, Bissell MJ (1995). The origin of the myofibroblasts in breast cancer. Recapitulation of tumor environment in culture unravels diversity and implicates converted fibroblasts and recruited smooth muscle cells. J Clin Invest.

[B6] Okada A, Bellocq JP, Rouyer N, Chenard MP, Rio MC, Chambon P, Basset P (1995). Membrane-type matrix metalloproteinase (MT-MMP) gene is expressed in stromal cells of human colon, breast, and head and neck carcinomas. Proc Natl Acad Sci U S A.

[B7] Nielsen BS, Sehested M, Timshel S, Pyke C, Dano K (1996). Messenger RNA for urokinase plasminogen activator is expressed in myofibroblasts adjacent to cancer cells in human breast cancer. Lab Invest.

[B8] Unden AB, Sandstedt B, Bruce K, Hedblad M, Stahle-Backdahl M (1996). Stromelysin-3 mRNA associated with myofibroblasts is overexpressed in aggressive basal cell carcinoma and in dermatofibroma but not in dermatofibrosarcoma. J Invest Dermatol.

[B9] Samoszuk M, Tan J, Chorn G (2005). Clonogenic growth of human breast cancer cells co-cultured in direct contact with serum-activated fibroblasts. Breast Cancer Res.

[B10] Bhowmick NA, Neilson EG, Moses HL (2004). Stromal fibroblasts in cancer initiation and progression. Nature.

[B11] Binda MM, Matar P, Gonzalez AD, Rozados VR, Gervasoni SI, Scharovsky OG, Bonfil RD (2002). Differential production of angiostatin by concomitant antitumoral resistance-inducing cancer cells. Int J Cancer.

[B12] Fidler IJ (1975). Biological behavior of malignant melanoma cells correlated to their survival in vivo. Cancer Res.

[B13] Scherer WF, Syverton JT, Gey GO (1953). Studies on the propagation in vitro of poliomyelitis viruses. IV. Viral multiplication in a stable strain of human malignant epithelial cells (strain HeLa) derived from an epidermoid carcinoma of the cervix. J Exp Med.

[B14] Kovacsovics-Bankowski M, Rock KL (1994). Presentation of exogenous antigens by macrophages: analysis of major histocompatibility complex class I and II presentation and regulation by cytokines. Eur J Immunol.

[B15] Thompson DL, Lum KD, Nygaard SC, Kuestner RE, Kelly KA, Gimble JM, Moore EE (1998). The derivation and characterization of stromal cell lines from the bone marrow of p53-/- mice: new insights into osteoblast and adipocyte differentiation. J Bone Miner Res.

[B16] Baba M, Obara T, Bonfil RD, Yamaguchi Y, Trump BF, Resau J, Klein-Szanto AJ (1988). Resistance to serum-induced terminal differentiation in normal human tracheobronchial epithelial cells after in vivo exposure to 7,12-dimethylbenz[a]anthracene. Jpn J Cancer Res.

[B17] Dong Z, Bonfil RD, Chinni S, Deng X, Trindade Filho JC, Bernardo M, Vaishampayan U, Che M, Sloane BF, Sheng S, Fridman R, Cher ML (2005). Matrix metalloproteinase activity and osteoclasts in experimental prostate cancer bone metastasis tissue. Am J Pathol.

[B18] Bonfil RD, Sabbota A, Nabha S, Bernardo MM, Dong Z, Meng H, Yamamoto H, Chinni SR, Lim IT, Chang M, Filetti LC, Mobashery S, Cher ML, Fridman R (2006). Inhibition of human prostate cancer growth, osteolysis and angiogenesis in a bone metastasis model by a novel mechanism-based selective gelatinase inhibitor. Int J Cancer.

[B19] Bonfil RD, Vinyals A, Bustuoabad OD, Llorens A, Benavides FJ, Gonzalez-Garrigues M, Fabra A (1994). Stimulation of angiogenesis as an explanation of Matrigel-enhanced tumorigenicity. Int J Cancer.

[B20] Deng T, Karin M (1993). JunB differs from c-Jun in its DNA-binding and dimerization domains, and represses c-Jun by formation of inactive heterodimers. Genes Dev.

[B21] Torii S, Yamamoto T, Tsuchiya Y, Nishida E (2006). ERK MAP kinase in G cell cycle progression and cancer. Cancer Sci.

[B22] Eferl R, Wagner EF (2003). AP-1: a double-edged sword in tumorigenesis. Nat Rev Cancer.

[B23] Shen Q, Uray IP, Li Y, Krisko TI, Strecker TE, Kim HT, Brown PH (2007). The AP-1 transcription factor regulates breast cancer cell growth via cyclins and E2F factors. Oncogene.

[B24] Camps JL, Chang SM, Hsu TC, Freeman MR, Hong SJ, Zhau HE, von Eschenbach AC, Chung LW (1990). Fibroblast-mediated acceleration of human epithelial tumor growth in vivo. Proc Natl Acad Sci U S A.

[B25] Shekhar MP, Pauley R, Heppner G (2003). Host microenvironment in breast cancer development: extracellular matrix-stromal cell contribution to neoplastic phenotype of epithelial cells in the breast. Breast Cancer Res.

[B26] Bissell MJ, Radisky DC, Rizki A, Weaver VM, Petersen OW (2002). The organizing principle: microenvironmental influences in the normal and malignant breast. Differentiation.

[B27] Allinen M, Beroukhim R, Cai L, Brennan C, Lahti-Domenici J, Huang H, Porter D, Hu M, Chin L, Richardson A, Schnitt S, Sellers WR, Polyak K (2004). Molecular characterization of the tumor microenvironment in breast cancer. Cancer Cell.

[B28] Atula S, Grenman R, Syrjanen S (1997). Fibroblasts can modulate the phenotype of malignant epithelial cells in vitro. Exp Cell Res.

[B29] DeCosse JJ, Gossens CL, Kuzma JF, Unsworth BR (1973). Breast cancer: induction of differentiation by embryonic tissue. Science.

[B30] Micke P, Ostman A (2005). Exploring the tumour environment: cancer-associated fibroblasts as targets in cancer therapy. Expert Opin Ther Targets.

[B31] De Wever O, Mareel M (2003). Role of tissue stroma in cancer cell invasion. J Pathol.

[B32] Boivin-Angele S, Pedron S, Bertrand S, Desmouliere A, Martel-Planche G, Lefrancois L, Bancel B, Trepo C, Marion MJ (2000). Establishment and characterization of a spontaneously immortalized myofibroblast cell line derived from a human liver angiosarcoma. J Hepatol.

[B33] Jones C, Nonni AV, Fulford L, Merrett S, Chaggar R, Eusebi V, Lakhani SR (2001). CGH analysis of ductal carcinoma of the breast with basaloid/myoepithelial cell differentiation. Br J Cancer.

[B34] Petersen OW, Nielsen HL, Gudjonsson T, Villadsen R, Rank F, Niebuhr E, Bissell MJ, Ronnov-Jessen L (2003). Epithelial to mesenchymal transition in human breast cancer can provide a nonmalignant stroma. Am J Pathol.

[B35] Walter-Yohrling J, Pratt BM, Ledbetter S, Teicher BA (2003). Myofibroblasts enable invasion of endothelial cells into three-dimensional tumor cell clusters: a novel in vitro tumor model. Cancer Chemother Pharmacol.

[B36] Kataoka H, Tanaka H, Nagaike K, Uchiyama S, Itoh H (2003). Role of cancer cell-stroma interaction in invasive growth of cancer cells. Hum Cell.

[B37] Sternlicht MD, Kedeshian P, Shao ZM, Safarians S, Barsky SH (1997). The human myoepithelial cell is a natural tumor suppressor. Clin Cancer Res.

[B38] Nguyen M, Lee MC, Wang JL, Tomlinson JS, Shao ZM, Alpaugh ML, Barsky SH (2000). The human myoepithelial cell displays a multifaceted anti-angiogenic phenotype. Oncogene.

[B39] Barsky SH, Karlin NJ (2005). Myoepithelial cells: autocrine and paracrine suppressors of breast cancer progression. J Mammary Gland Biol Neoplasia.

[B40] Shi HY, Zhang W, Liang R, Kittrell F, Templeton NS, Medina D, Zhang M (2003). Modeling human breast cancer metastasis in mice: maspin as a paradigm. Histol Histopathol.

